# A proposed modulatory role of the endocannabinoid system on adipose tissue metabolism and appetite in periparturient dairy cows

**DOI:** 10.1186/s40104-021-00549-3

**Published:** 2021-03-05

**Authors:** Madison N. Myers, Maya Zachut, Joseph Tam, G. Andres Contreras

**Affiliations:** 1 Department of Large Animal Clinical Sciences, College of Veterinary Medicine, Michigan State University, East Lansing, MI 48824 USA; 2 Department of Ruminant Science, Institute of Animal Sciences, Agricultural Research Organization / Volcani Center, 7505101 Rishon LeZion, Israel; 3 Obesity and Metabolism Laboratory, The Institute for Drug Research, School of Pharmacy, Faculty of Medicine, The Hebrew University of Jerusalem, 9112001 Jerusalem, Israel

**Keywords:** Adipogenesis, Adipose tissue, Dairy cow health, Endocannabinoids, Endocannabinoid system, Lipogenesis, Lipolysis

## Abstract

To sustain the nutrient demands of rapid fetal growth, parturition, and milk synthesis, periparturient dairy cows mobilize adipose tissue fatty acid stores through lipolysis. This process induces an inflammatory response within AT that is resolved as lactation progresses; however, excessive and protracted lipolysis compounds the risk for metabolic and inflammatory diseases. The suppression of lipolytic action and inflammation, along with amplification of adipogenesis and lipogenesis, serve as prospective therapeutic targets for improving the health of periparturient dairy cows. Generally, the activation of cannabinoid receptors by endocannabinoids enhances adipogenesis and lipogenesis, suppresses lipolysis, and increases appetite in mammals. These biological effects of activating the endocannabinoid system open the possibility of harnessing the endocannabinoid system through nutritional intervention in dairy herds as a potential tool to improve dairy cows’ health, although much is still to be revealed in this context. This review summarizes the current knowledge surrounding the components of the endocannabinoid system, elaborates on the metabolic effects of its activation, and explores the potential to modulate its activity in periparturient dairy cows.

## Introduction

**Fig. 1:**
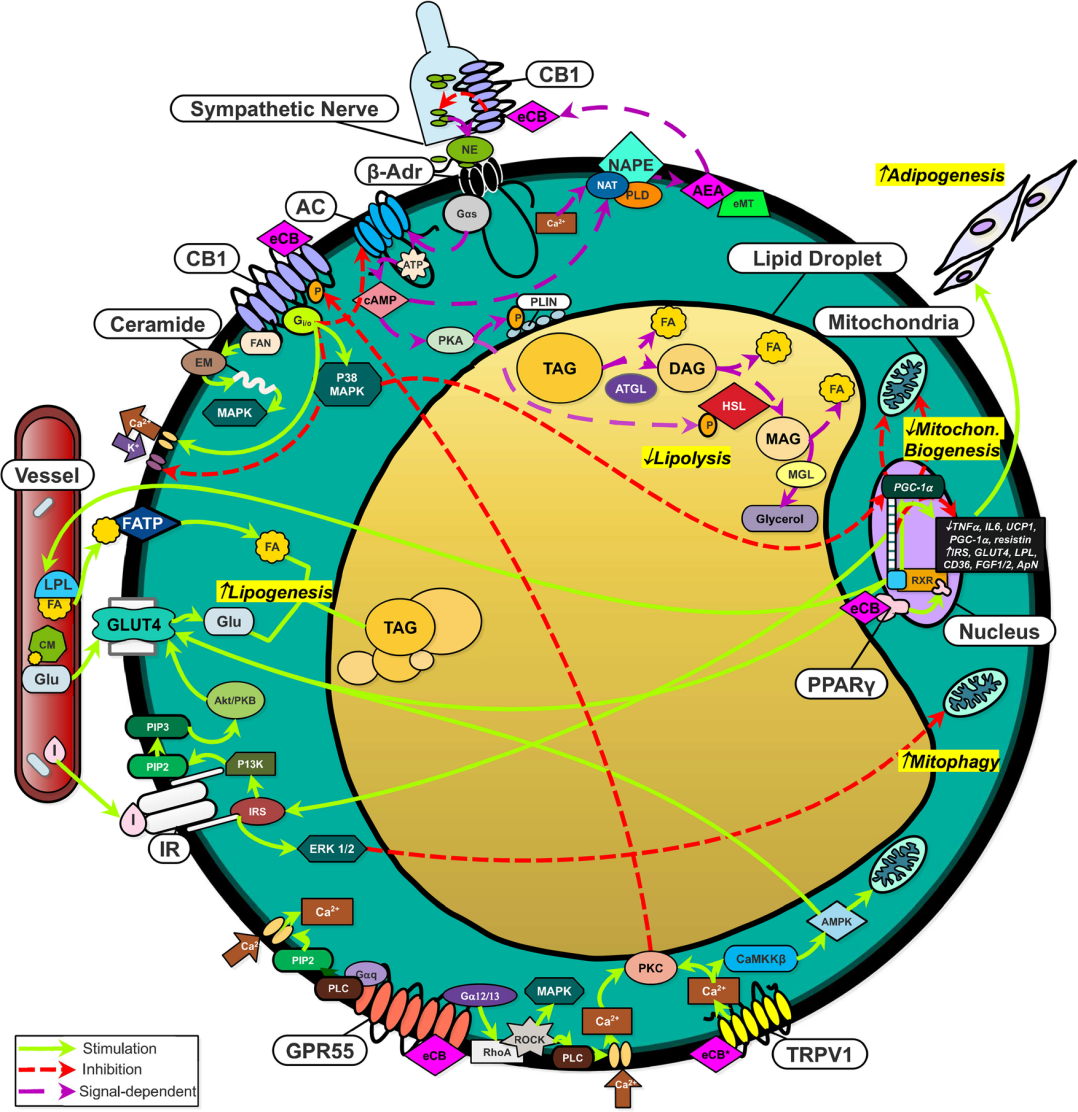
The ECS promotes energy conservation and reduces lipolysis in mature white adipocytes. Lipolysis: CB1 activation on the adipocyte surface inhibits the activity of the lipolytic enzymes HSL and PLIN, while CB1 activation in autonomous nerves limits the release of catecholamines, the primary ligand for β-adrenergic receptors. Lipogenesis and adipogenesis: eCBs bind and activate PPARγ directly, enhancing transcription of pro-lipogenic and pro-adipogenic genes: Improved expression of GLUT4 and LPL leads to greater levels of lipogenesis. Mitochondrial biogenesis: CB1 activation suppresses the activity of the transcriptional coactivator PGC-1α; the principal inducer of mitochondrial biogenesis. Abbreviations: 5′ AMP-activated protein kinase (AMPK), Adenosine triphosphate (ATP), Adenylyl cyclase (AC), Adipose triglyceride lipase (ATGL), Anandamide (AEA), Beta adrenergic receptor (β-ADR), Calcium (Ca^2+^), Calcium/calmodulin-dependent protein kinase kinase 2 (CaMKKβ), Cannabinoid receptor 1 (CB1), Chylomicron (CM), Cyclic adenosine monophosphate (camp), Diacylglycerol lipase (DAG), Endocannabinoid membrane transporter (EMT), Extracellular signal-regulated kinase (ERK1/2), Factor associated with neutral sphingomyelinase activation (FAN), Fatty acid (FA), Fatty acid transport protein (FATP), G alpha subunit (Gαs), G protein-coupled receptor 55 (GPR55), Gi protein subunit o (G_i/o_), Glucose (GLU), Glucose transporter type 4 (GLUT-4), Guanine nucleotide binding protein subunit 12/13 (Gα12/13), Guanine nucleotide binding protein subunit q (GαQ), Hormone-sensitive lipase (HSL), Insulin (I), Insulin receptor (IR), Lipoprotein lipase (LPL), Mammalian target or rapamycin complex 1 (MTORC1), Mitogen-activated extracellular signal-regulated kinase (MEK), Mitogen-activated protein kinase (MAPK), Monoacylglycerol (MAG), Monoglyceride lipase (MGL), N-Acyltransferase (NAT), NAPE-phospholipase D (PLD), N-Arachidonyl phosphatidylethanolamine (NAPE), Neutral sphingomyelinase (EMN), Norepinephrine (NE), Perilipin (PLIN), Peroxisome proliferator-activated receptor gamma (PPARγ), Phosphatidylinositol 4,5 bisphosphate (PIP2), Phospholipase C (PLC), Phosphorylation (P), Potassium (K^+^), PPAR-gamma coactivator 1α (PGC-1α), Protein kinase A (PKA), Protein kinase B (Akt/PKB), Protein kinase C (PKC), Ras homolog family member A (RHOA), Retinoid X receptor (RXR), Rho-associated protein kinase (ROCK), Sphingomyelin (EM), Triacylglycerol (TAG), Tuberous sclerosis complex 2 (TSC2), Vallinoid receptor 1 (TRPV1)

To meet the energy needs of her growing fetus, parturition, and onset of lactation during a dairy cow’s periparturient period, profound metabolic and endocrine adaptations must occur. It is well established that this period of high-energy requirement is coupled with a reduction in appetite (i.e., dry matter intake [1]), setting the stage for negative energy balance. To compensate for energy deficits, fatty acids (FA) stored as triacylglycerols (TAG) within adipose tissue’s (AT) cellular unit, the adipocyte, are mobilized (Fig. 1) [2]. Although this metabolic challenge is normal and necessary during the periparturient period, some cows fail to adapt, which increases their risk for metabolic and inflammatory diseases.

**Fig. 2:**
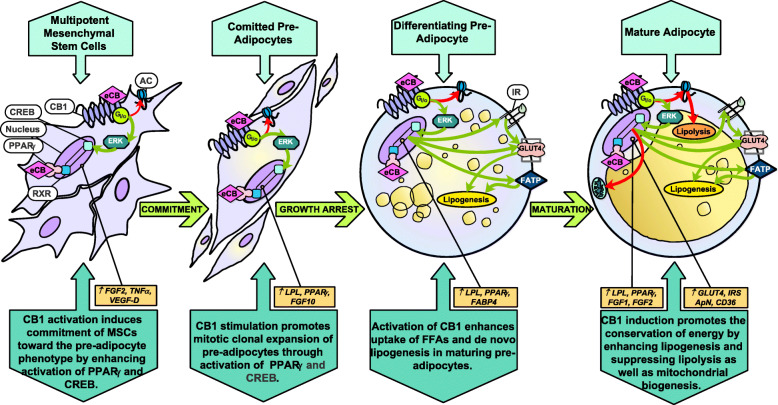
The ECS enhances transcriptional machinery of PPARγ and lipid accumulation, promoting adipogenesis and lipogenesis in maturing progenitor cells. Red arrows: inhibitory effect downstream of eCB stimulation. Green arrows: stimulatory effect downstream of eCB stimulation. Abbreviations: Adenylyl cyclase (AC), adiponectin (ApN), Cannabinoid receptor 1 (CB1), Cyclic adenosine monophosphate response element-binding protein (CREB), Endocannabinoid (eCB), Extracellular signal-regulated kinase (ERK), Fatty acid binding protein 4 (FABP4), Fatty acid translocase (CD36), Fatty acid transport protein (FATP), Fibroblast growth factor 1, 2, 10 (FGF1, 2, 10), Free fatty acid (FFA), Gi protein subunit o (G_i/o_), Glucose transporter type 4 (GLUT-4), insulin (I), Insulin receptor (IR), Insulin receptor substrate (IRS), Lipoprotein lipase (LPL), Peroxisome proliferator-activated receptor gamma (PPARγ), Retinoid X receptor (RXR), Tumor necrosis factor alpha (TNFα), Vascular endothelial growth factor D (VEGF-D)

The AT is composed of adipocytes, fibroblasts, progenitor cells, endothelial cells, and immune cells. White adipocytes are largely comprised of a single substantial fat droplet (up to 90% of the cell’s volume), a limited number of mitochondria, and a compressed nucleus. In response to increased energy needs, TAG stored in the lipid droplet are hydrolyzed during lipolysis to release FA and glycerol. In times of excess energy, the AT expands by enlarging the size of adipocytes’ lipid droplets (lipogenesis), or by increasing the number of adipocytes (adipogenesis) (as displayed in Figs. 1 and 2). In addition to the provision of energy, AT serves as an endocrine organ and secretes several factors associated with the modulation of energy metabolism, including adipokines (e.g., adiponectin, leptin, resistin) and, as recently described, endocannabinoids (eCBs) [3]. Research over the past two decades highlights the endocannabinoid system (ECS) as a potent coordinator of AT function. The ECS consists of eCBs, cannabinoid receptors, and enzymes involved in the synthesis and degradation of eCBs. Functions of the ECS include regulation of physical exertion, immunomodulation, modification of cellular proliferation, and preservation of energy-storing reservoirs [4]. The ECS, when active, favors the accumulation of fat mass through both central and peripheral pathways [5]. Within AT, ECS activation promotes adipogenesis and lipogenesis and impedes lipolytic activity (Figs. 1 and 2). In addition to these functions, the ECS enhances appetite in mammals through paracrine and endocrine signals as well as neural pathways. The role of ECS on modulating these important metabolic processes emphasizes the potential of the ECS to reduce the intensity and duration of negative energy balance in periparturient cows.

### The adipose tissue endocannabinoid system

#### Endocannabinoids

eCBs consist of lipid intermediaries, including amides, esters, and ethers of polyunsaturated FAs [6]. eCBs possess structural similarities to exogenous cannabinoids, such as a phenolic hydroxyl at the carbon C-1 and an alkyl side chain at the carbon C-3 [7]. Both eCBs and exogenous CBs (such as (−)-*trans*-Δ^9^-tetrahydrocannabinol, THC) bind to CB receptors in mammalian tissues [8].

Synthesized rapidly in response to an increase in intracellular calcium levels, metabolic stress, or cellular damage, eCBs are derived from dietary FAs [9]. These ligands bind and activate the canonical CB receptors type 1 and 2 (CB1 and CB2, respectively), the vallinoid receptor TRPV1, G protein-coupled receptor 55 (GPR55), and members of the peroxisome proliferator-activated receptor (PPAR) family [10]. The two most abundant (and potent) eCBs are the arachidonic acid-containing AEA (anandamide, *N*-arachidonoylethanolamide) and 2-AG (2-arachidonyglycerol) [11]. Lesser-known eCB molecules include palmitoylethanolamine (PEA) and oleoylethanolamine (OEA). These compounds have not been studied in ruminants and, therefore, are beyond the scope of this review (readers are referred to a detailed literature revision elsewhere [12]).

In dairy cows, plasma 2-AG concentrations increase from 1.5 ± 0.94 nmol/mL during the dry period to 3.0 ± 0.94 nmol/mL one month after parturition [13]. In AT from cows exhibiting high postpartum weight loss (> 8% during the first month postpartum), AEA and 2-AG levels double from 0.94 ± 0.23 fmol/mg and 0.56 ± 0.10 nmol/mg at − 14 days relative to parturition, to 2.18 ± 0.23 fmol/mg and 0.97 ± 0.10 nmol/mg 4 days after calving [14]. In contrast, cows exhibiting low weight loss do not show dramatic increases in plasma and AT AEA or 2-AG levels when compared to high weight loss groups [13, 14]. These changes in plasma and AT eCB content suggest that the ECS may be activated to a greater extent in cows experiencing greater levels of lipolysis. It is presently unknown if high eCB content is an anti-lipolytic response of the AT to reduce TAG breakdown or if it is a consequence of the high availability of eCB precursors driven by lipolysis.

### Biosynthesis of eCBs

#### AEA

**Fig. 3:**
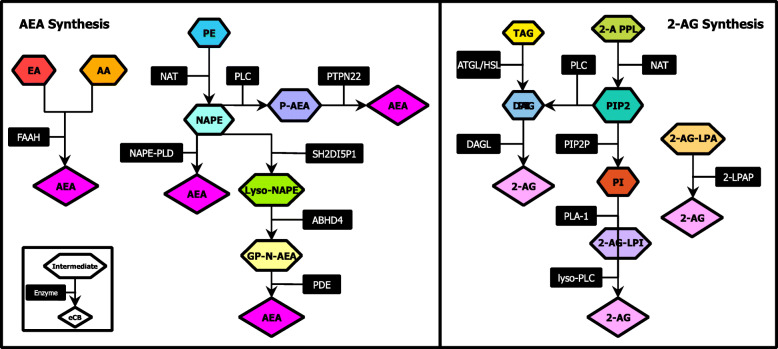
Proposed biosynthetic pathways for AEA (**a**) and 2-AG (**b**) formation. Abbreviations: Arachidonic acid (AA), Arachidonoyl ethanolamine (AEA), Ethanolamine (EA), Fatty acid amide hydrolase (FAAH), Glycerophosphoanandamide (GP-N-AEA), Lyso-NAPE, abhydrolase domain-containing 4 (ABHD4), N-acyltransferase (NAT), NAPE-phospholipase D (NAPE-PLD), N-arachidonylphosphatidylethanolamine (NAPE), Phosphatidyl ethanolamine (PE), Phosphoanandamide (P-AEA), Phosphodiesterase (PDE), Phospholipase C (PLC), Protein tyrosine phosphatase (PTPN22), Src Homology 2 domain-containing inositol-5-phosphatase 1 (SH2DI5P1). 2-AG lysophosphatidyl inositol (2-AG-LPI), 2-arachidonoyl glycerol (2-AG), 2-arachidonoyl phospholipids (2-A PPL), 2-lysophosphatidic acid phosphatase (2-LPAP), Adipose triglyceride lipase (ATGL), Diacylglycerol (DAG), DAG lipase (DAGL), Hormone-sensitive lipase (HSL), Lyso-PLC, 2-AG-lysophosphatidic acid (2-AG-LPA), N-acyltransferase (NAT), Phosphatidyl inositol (PI), Phosphatidyl inositol bisphosphate 2 (PIP2), Phospholipase 1 (PLA1), PIP2 phosphatase (PIP2P), Triacylglycerol (TAG)

There are three proposed biosynthetic pathways for AEA (illustrated in Fig. 3a): The first pathway begins with the *N-*acylation of the phospholipid membrane precursor phosphatidylethanolamine (PE) by the Ca^2+^-dependent *N-*acyltransferase (NA) to produce *N-*arachidonylphosphatidylethanolamine (NAPE) [15, 16]. Next Ca^2+^-activated enzyme *N*-arachidonylphosphatidylethanolamine-specific phospholipase D (NAPE-PLD) acts on NADE yielding AEA. The second pathway includes the hydrolysis of NAPE (after *N-*acylation by NA) by type-C phospholipase (PLC) to phosphoanandamide. This is followed by dephosphorylation by Src homology 2 domain-containing inositol-5-phosphatase 1 to produce AEA [17]. In pathway three, repeated hydrolytic cleavage of NAPE’s acyl groups by the serine hydrolase abhydrolase domain containing 4 to form lyso-NAPE and then glycerophospho-*N*-AEA. This product is then hydrolyzed by the metal-dependent glycerophosphodiester phosphodiesterase 1 to AEA [18].

#### 2-AG

2-AG is the hydrolyzed product of 2-arachidonoyl-containing phospholipids (mainly arachidonoyl-containing phosphatidyl inositol bis-phosphate, PIP2). There are three main routes proposed for the intracellular biosynthesis of 2-AG (Fig. 3b): 1) diacylglycerol (DAG) is synthesized from PIP2 and then hydrolyzed by the enzyme diacylglycerol lipase (DAGL). 2) 2-arachidonoyl-lysophosphatidic acid is hydrolyzed by 2-lysophosphatidic acid phosphatase into the bioactive eCB. 3) 2-arachidonoyl-lysophosphatidylinositosol, a derivative of PIP2, is synthesized into 2-AG by lyso-PLC [19].

### Degradation of eCBs

#### Non-oxidative enzymatic degradation of eCBs

The two primary enzymes that catabolize eCBs are the serine hydrolase FAAH and monoacylglycerol lipase (MAGL) [20]. FAAH, a membrane-bound enzyme, favors AEA as a substrate but hydrolyzes other long-chain amides and amines (detailed description in [21, 22]. The hydrolysis of AEA, OEA, and PEA by FAAH yields ethanolamine and AA, oleic acid, and palmitic acid, respectively [23]. The expression and activity of these non-oxidative pathways in AT are affected by anatomical location and degree of adiposity. Visceral AT exhibits a higher expression of these eCB catabolic pathways compared to subcutaneous depots [24]. In humans, levels of visceral FAAH are lower in obese humans compared to lean subjects [25], suggesting that eCB degradation by FAAH may be inhibited in larger adipocytes.

MAGL hydrolyzes 2-AG and is likely to be the primary route of degradation for 2-AG into AA and glycerol [26]. Unlike FAAH, which is expressed ubiquitously, MAGL is expressed predominantly in AT [27]. Notably, genetic or pharmacological blockade of MAGL in mice leads to elevated levels of 2-AG in the brain and enhances the sensitivity of CB1 to eCBs [20], unlike FAAH blockade, which causes CB1 desensitization [28].

MAGL appears to be a major degradative enzyme of eCBs in periparturient dairy cows. We demonstrated that MAGL protein abundance in AT is lower in cows experiencing high weight loss compared to those with minimal body weight changes [14]. In the same study, FAAH concentrations remained stable across groups throughout the sampling period [14]. However, MAGL protein abundance was higher in insulin-resistant vs. insulin-sensitive AT from postpartum cows [29]. More information on the role of MAGL in AT lipolysis and ECS activation in dairy cows is required to explore the possibility of using this enzyme as a pharmacological target for intervention.

Additional enzymes known to degrade eCBs include the serine hydrolases α/β hydrolase 6 (ABHD6), and α/β hydrolase 12 (ABHD12), which degrade 2-AG in the brain [30], and *N*-acylethanolamine-hydrolyzing acid amidase (NAAA), which hydrolyzes *N*-acylethanolamines under acidic conditions [31]. In dairy cows, the expression of genes encoding for ABHD12 and NAAA was reported in the reproductive tract but not in AT. Periparturient dairy cows with subclinical endometritis had a reduced endometrial mRNA expression of *NAAA* compared to healthy controls [32]. *ABHD6* and *ABHD12* levels are expressed differently during the follicular development of oocytes [33]; however, the role that these enzymes play in the ECS in AT remains to be explored in dairy cows.

#### Oxidative degradation of eCBs

Enzymes such as cyclooxygenases (COX), lipoxygenases (LOX), and cytochromes P450 (CYP450) are capable of oxidizing eCBs (reviewed in [34]). These enzymes are part of the inflammatory process as a source of inflammatory lipid mediators, also known as oxylipids. These products are oxidized FAs derived from phospholipid membranes or triglycerides contained in lipid droplets that regulate the different stages of inflammation from onset to resolution [35]. In AT from dairy cows, we demonstrated the expression and functionality of COX, LOX, and P450s [36]. The full extent to which these oxidative enzymes act on eCBs in ruminant AT remains unknown. However, pharmacological blockade of these eCB-oxidizing pathways in dairy cows’ AT may be beneficial during the periparturient period as eCBs may promote lipogenesis and thus counteract lipolysis. Also, there are reports of eCBs acting as suppressors of AT inflammation [37] and inhibitors of pain sensation [38]; therefore, inhibiting eCB-degrading enzymes could offer some advantages by reducing AT inflammation. Nevertheless, recent studies indicate that when eCB precursors are fed at high levels in mice and salmon, inflammatory responses within AT are observed along with enhanced levels of eCBs [39]. These conflicting findings highlight the complexity of the ECS in AT and emphasize the need for further research to elucidate the role of eCB in AT inflammatory responses of dairy cattle.

### Cellular receptors of eCBs

#### CB1

The two primary receptors of the ECS are the G protein-coupled receptors (GCPRs) CB1 and CB2, encoded by the *CNR1* and *CNR2* genes, respectively. CB1 belongs to the Class A GCPRs, known to activate inward-propelling potassium channels and inhibit calcium channels [40]. In humans and rodents, CB1 receptors are expressed at the highest levels in neural tissue, and lower levels of expression are observed in AT, liver, skeletal muscle, and peripheral organs [41]. In adipocytes, CB1 receptor stimulation increases the uptake of glucose and lipogenesis while inhibiting lipolysis [42]. CB1 conducts its response via G protein G_i/o_-mediated reduction in adenylate cyclase action, suppressing the activation of hormone-sensitive lipase (HSL) through the halt in cyclic adenosine monophosphate (cAMP) production [43].

#### CB2

CB2 is found primarily in microvascular endothelial cells and on the surface of immune cells, most commonly those derived from the hematopoietic lineage [44]. CB2 is known to exert anti-inflammatory effects in peripheral tissues [45], and only low levels of CB2 expression are detected in monogastric AT [46]. In humans, CB2 expression has been shown to decrease as pre-adipocytes mature into adipocytes *in vitro* [24]. This mRNA expression pattern suggests that within AT, CB2 content is higher in preadipocytes, macrophages, and vascular cells rather than in mature adipocytes [24].

#### GPR55

Belonging to the rhodopsin-like (Class A) family of GPCRs, GPR55 expression is detected in brain and neural tissues, immune cells, spleen, blood vessels, small intestine, endometrium, and AT [47]. This receptor activates PLC, which stimulates inward-propelling Ca^2+^ channels. PLC activation catalyzes the cleavage of PIP2 to inositol triphosphate and DAG (diacylglycerol) [48]; GPR55 activation also promotes insulin secretion in pancreatic β-cells [49], and its suppression is associated with adiposity [50] and impaired insulin signaling in AT [47].

#### TRPV1

TRPV1 is a non-selective ion channel expressed most prominently in neural tissues. This receptor is also found in a wide range of tissues, including blood vessels, the gastrointestinal tract, immune cells, endothelial cells lining the urinary tract, and adipocytes [51]. TRPV1’s direct role in AT metabolism lies in its ability to increase intracellular calcium levels, which activates mitochondrial biogenesis through enhanced 5′ adenosine monophosphate-activated protein kinase (AMPK) activity [52] and by peroxisome proliferator-activated receptor gamma coactivator 1 (PGC-1α) activation of uncoupling protein 1 (UCP-1) by route of sirtuin-1 (SIRT-1) [53]. The eCB AEA is known to activate the TRPV1 receptor and B-lymphocyte-derived (B1 cell-derived) leukotriene B4, which regulates the local inflammatory response in AT as a TRPV1 agonist [54].

#### CB receptors in AT of dairy cows

AT expression of *CNR1* (encoding CB1) and *CNR2* (encoding CB2) increase after calving in dairy cows exhibiting high rates of lipolysis [14]. Dirandeh et al. [55] demonstrated that higher gene expression of *CNR2, NAPEPLD,* and *FAAH* in AT coincides with enhanced expression of pro-inflammatory genes (*TNF-α, IL-6, IL-1β*) at 21 and 42 days postpartum in cows exhibiting intense AT lipolysis. Also, CB receptor expression is affected during inflammatory diseases, as shown in cows with endometritis, where endometrial *CNR2* transcription is amplified [32]. These expression patterns indicate a possible link between ECS and AT inflammatory responses; however, further research is required to understand the role of ECS in adipose inflammation in dairy cows, specifically during the periparturient period when AT undergoes dramatic lipolysis and remodeling [56].

### The ECS modulates adipogenesis and lipid mobilization in AT

#### Alteration of adipogenesis via the ECS

Adipogenesis defines the determination and terminal differentiation of adipose progenitor cells into adipocytes. De novo adipogenesis enhances the capacity of AT for storing energy when present adipocytes reach their maximum volume during positive energy balance (i.e., hyperplasia) [57]. Since adipogenesis reduces lipotoxicity, promoting the differentiation of new adipocytes may be effective in reducing the deleterious effects of excessive lipolysis in periparturient cows.

PPARγ is the master regulator of adipogenesis, although other PPARs – α, β/δ – contribute as well [58]. Three variants of PPARγ have been identified; the most prominent isoform is PPARγ_2_ as it is expressed primarily in AT and is heavily involved in energy homeostasis [42]. Upon activation by eCBs (or other ligands), the PPAR family binds to the retinoid X receptor (RXR) to form heterodimers. This complex then binds to DNA response elements, triggering the expression of adipogenic and lipogenic gene networks (Figs. 1 and 2) [59].

During the differentiation of adipocyte progenitor cells, expression of CB1, NAPE-PLD, and DAGL increase, while expression of the eCB-degradative enzymes FAAH, MAGL, and NAAA decrease [60]. CB1 and CB2 are present in adipocyte progenitor cells [61] and, as illustrated in Fig. 2, CB1 stimulation enhances the capacity of adipose stem cells to commit to preadipocytes through downstream activation of the cAMP response element-binding protein (CREB) and PPARγ_2_ [62]. Although CB1 stimulation decreases intracellular levels of cAMP [63], phosphorylation of CREB may occur through G_i/o_ activation of ERK [64]. CREB binds to adipogenic promoters such as FABP, FAS, and C/EBPβ. This enhances the adipogenic commitment cascade in progenitor cells. PPARγ_2_’s adipogenic activity, on the other hand, is directly improved by the binding of eCBs and CREB [65].

When murine 3 T3-F442A preadipocytes are treated with the CB1 stimulant HU210, these cells show enhanced expression of PPARγ_2_ and adiponectin and increase the number of lipid droplets formed [66]. In human preadipocytes, CB1 stimulation promotes glucose uptake through increased intracellular Ca^2+^ mobilization from the endoplasmic reticulum and insulin-dependent phosphatidylinositol 3 kinase (PI3K)/AKT (Protein Kinase B) pathway activation (see Fig. 1) [24]. CB1 inhibition with the selective agent Rimonabant (SR141716A) in co-incubation with CB1 activators was shown to mitigate adipogenesis via inhibition of p42/44 mitogen-activated protein kinase (MAPK) activity. In the same type of cells, adiponectin levels and glyceraldehyde-3-phosphate dehydrogenase activity increases during exposure to CB1 agonists, expounding the significance of CB1 stimulation in both adipogenesis and lipogenesis [67].

To our knowledge, there are no studies evaluating AT adipogenic responses during ECS activation in dairy cows. However, in periparturient cows exhibiting moderate to high body weight loss, the gene expression of N*APEPLD* and *PPARγ* is upregulated and positively associated [55], suggesting a possible physiological response as AEA synthesized by NAPE-PLD is capable of activating PPARγ, enhancing adipogenesis directly.

#### Lipogenesis is amplified by ECS activation

In ruminants, de novo lipogenesis is the biological process by which neutral lipids and phospholipids (i.e., TAG, phospholipids, cholesterol, or sphingolipids) are biosynthesized from dietary volatile FAs within the cytoplasm of adipocytes or hepatocytes. A key stimulator of lipogenesis is insulin. This pancreatic peptide promotes glucose transporter type 4 (GLUT-4) and lipoprotein lipase (LPL) activity. In monogastric animals, upon ligand binding to CB1, lipogenesis is enhanced by three mechanisms: 1) the promotion of LPL activity; 2) the inhibition of AMPK; and 3) the augmentation of insulin-dependent glucose uptake. LPL hydrolyzes TAG found in circulating plasma lipoproteins and thus increases FA available for lipogenesis. In preadipocytes and adipocytes, LPL transcription and activity are heavily regulated by insulin. Glucose, on the other hand, glycosylates LPL intended for secretion from adipocytes to capture TAG and enhances LPL synthesis within adipocytes [68]. eCB binding to CB1 increases expression and activity of LPL [69]; however, the mechanism by which this occurs is unknown.

CB1 activation triggers G_i/o_-dependent signaling pathways [63], enhancing the excretion of intracellular calcium into the extracellular matrix. This intracellular Ca^2+^ reduction hinders AMPK action via Ca^2+^/calmodulin-dependent protein kinase kinase β (CaMKKβ) [70]. AMPK, a serine/threonine protein kinase, regulates energy homeostasis by enriching pathways that generate ATP and diminishing energy-consuming pathways [71]. AMPK decreases FA synthesis by reducing ACC activity and, subsequently, malonyl-CoA availability. AMPK regulation occurs through mechanisms directly related to hindered cAMP production (G_i/o_-dependent signaling pathways triggered by CB1 activation, for example), reductions of intracellular calcium, and elevations of the concentrations of ATP. G_i/o_ pathway activation also hinders the action of adenylate cyclase, most likely as a means to conserve ATP [63].

Intracellular glucose levels increase with CB1 activation [24]. Downstream of CB1, PI3K/AKT pathway stimulation promotes the translocation of glucose transporters to the cell surface from the cytoplasmic vesicles. This explains the increase in insulin-dependent glucose transport into the cell that has been stimulated with eCBs [72]. Like CB1, GPR55 is a positive regulator of insulin action and enhances levels of intracellular glucose in an insulin-dependent manner [47].

In dairy cows with intense body weight loss, AT C*NR1* expression is greater than in those maintaining their body condition, suggesting that the eCB receptor CB1 activation is a response to increased free FA content in AT [14]. However, more information is required on the specific mechanisms that govern the ECS in periparturient cows in order to fully understand its involvement in the regulation of lipogenesis.

#### The ECS suppresses lipolysis

Lipolysis occurs during negative energy balance when stored TAG are broken down into free FAs and glycerol. Stimulation of TAG catabolism occurs upon binding of hormones (glucagon, growth hormone) and bioactive amines (adrenaline, norepinephrine) to either β-adrenergic or glucagon receptors, triggering the c-AMP-dependent lipolytic cascade via protein kinase A (PKA) phosphorylation of HSL and perilipin [73]. Within the adipocyte, TAG are hydrolyzed by adipose triglyceride lipase (ATGL) into diacylglycerol (DAG). From this point, DAG is hydrolyzed to monoacylglycerol (MAG) by HSL. Once broken down into free FAs and glycerol by MAGL, FA transport proteins (FAT/CD36, FATP1, FABP) can direct FAs to the mitochondria for oxidation or the RER where FA can be re-esterified into TAG or released into the vasculature surrounding the adipocyte for transport (extensively reviewed in [74]). Free FAs are then used as energy substrates primarily by cardiac, renal, or muscular tissues, but may also be utilized by most organs (exceptions being erythrocytes and the renal medulla).

The anti-lipolytic effect of CB1 stimulation in adipocytes occurs through G_i/o_ inhibition of cAMP production, which limits the downstream phosphorylation of HSL and perilipin [75]. Transcriptional effects downstream of CB1 activation in AT include the suppression of lipolysis-associated enzymes (carnitine-acyl-CoA transferase, carnitine palmitoyltransferase 2, and crotonase), along with downregulation of β-adrenergic and growth hormone receptor expression [76]. CB1 stimulation in AT sympathetic nerves suppresses catecholamine release, subsequently downregulating HSL activity through the decrease in cAMP produced by AC, which is no longer activated by β-adrenergic receptors [77] (Figs. 1 and 2).

Homeorhetic conditions in periparturient dairy cows drastically increase lipolysis around the time of parturition [78], yet excessive lipolysis leads to high disease incidence, reduced milk yield, and impaired reproductive performance. Considering the anti-lipolytic effects that follow CB1 stimulation and their potential benefits, the role of this receptor and the ECS should be further explored in periparturient cows.

### The ECS modulates mitochondrial function in AT

Despite the limited number and relative mass of mitochondria in white adipocytes, their role in AT homeostasis and remodeling is significant. Based on microenvironmental signals, mitochondria oxidize FAs and carbohydrates in the tricarboxylic acid cycle, and the subsequent electron transport chain provides ATP to the cell [79]. In addition to the provision of energy, mitochondria also play a key role in the differentiation of preadipocytes into adipocytes. In fact, mitochondrial FA metabolism and production of reactive oxygen species (ROS) are necessary to initiate adipogenesis [80]. This may be attributed to ROS’ role in insulin signaling or its ability to enhance PPARγ’s transcriptional machinery [81].

In line with its lipogenic effects, CB1 stimulation has been shown to limit mitochondrial biogenesis and increase mitophagy in AT (as illustrated in Figs. 1 and 2). These effects may reduce mitochondrial oxidaton of FA and possibly redirect these energy molecules to reesterification into triglycerides in the endoplasmic reticulum. Both genetic (*CNR1* knockout mice) and pharmacological (SR141716-treated mice) blockade of the CB1 receptor increased eNOS-dependent (endothelial nitric oxide synthase) mitochondrial biogenesis and AMPK activity in white adipocytes [82]. In another study, CB1 stimulation was associated with a decrease in PGC-1α expression, which corresponded to a direct decrease in mitochondrial mass and function [83].

Other molecules associated with suppressed mitochondrial function are ceramides [84]. These bioactive lipids are produced from a FA and sphingosine (de novo synthesis) or by the hydrolysis of sphingomyelin [85]. In monogastric AT, ceramides are known regulators of insulin signaling, inflammation, and intracellular metabolism [86]. Both CB1 and CB2 activation cause increases in intracellular ceramide production, likely a result of improved de novo ceramide synthesis through upregulation of the MAPKs extracellular signal-regulated kinase (ERK1/2), P38MAPK, and/or c-Jun N-terminal kinases (JNK) [87]. Heightened intracellular ceramide levels are associated with changes in mitochondrial membrane potential, the formation of new ion channels [88], and alterations in trifunctional proteins which are known to catalyze chain shortening reactions in mitochondrial FA β-oxidation [89].

Increased plasma ceramide in periparturient cows is positively associated with plasma acylcarnitine accretion, suggesting that FAs are partitioned away from β-oxidation in the mitochondria and toward the synthesis of sphingolipids [90]. The role of ceramide and sphingolipid biology in the metabolism of dairy cows is extensive (reviewed in [91]); however, it is currently unknown whether ceramides activate CB1 or CB2 in AT of dairy cows, and this warrants further investigation.

### The ECS modulates inflammatory responses

The effects of ECS activation on inflammatory responses are complex and vary depending on the tissue, eCBs, and the type of receptor. For example, AEA has anti-inflammatory effects, including the inhibition of chemoattractant cytokines secretion, especially those released at the early stages of the inflammatory process such as IL-6, IL-8, and MCP-1, along with completely blocking lipopolysaccharide (LPS)-triggered activation of NF-kappa β pathway in periodontal tissues [92]. As for the receptors, activation of the CB1 suppresses the proliferation of cells of the adaptive immune system, especially T-cells [93]. Blocking CB1 activity has been shown to increase LPS-mediated inflammation in the gut [94]. CB2 activation in immune cells inhibits the release of pro-inflammatory cytokines [95] and therefore prevents leukocyte migration and adhesion in the brain [96]. Nevertheless, there are reports indicating that inhibition of ECS receptor activity causes inflammatory responses. Blocking of CB1 with Rimonabant in the presence of LPS decreases expression and secretion of the pro-inflammatory cytokines TNFα and IL-6 in adipocytes [97]. Therefore, more research is required to fully elucidate the effects of eCBs and the activation of CB1 and CB2 receptors on inflammation in different tissue types.

In dairy cows, there are reports indicating that ECS activation may be indicative of subclinical inflammatory diseases [32, 98]. For example, periparturient cows with subclinical endometritis had reduced expression (2- to 4- fold) of markers of eCB degradation (*NAAA, FAAH*) compared to healthy controls. At the same time, these animals had higher expression (2.5- to 4-fold) of components of the eCB synthesis pathways, and ECS markers (*NAPEPLD,* CNR2) compared to controls [32]. These findings indicate that the ECS is activated to a greater extent in cows experiencing uterine inflammation post-calving [14]. Remarkably, ECS activity in the reproductive tract appears to be modified, at least at the gene expression level, by nutritional interventions, as reported by Abolghasemi et al. [99]. In their study, conjugated linoleic acid supplementation (75 g/d) from days 21 to 42 after parturition reduced the expression of uterine *CNR2* and *NAPEPLD .*

The interactions between ECS activation and inflammatory responses in AT of periparturient dairy cows is poorly characterized. However, we demonstrated that AT inflammation is triggered by high lipolysis rates postpartum, and this alters the activity of some components of the ECS system [14, 36]. Cows with high lipolysis have enhanced expression of components of the eCB biosynthetic pathways and ECS receptors, including NAPEPLD, CNR1, and CNR2 compared to periparturient cows with low lipolysis [14]. AT inflammation and lipolysis postpartum also affects the expression of eCBs degrading enzymes. Expression of *ALOX5 and ALOX15* (encoding for 5- and 15-lipoxygenase, respectively*)*, which are capable of metabolizing eCBs and subsequently produce oxylipid mediators of inflammation, varies throughout the periparturient period and also between cows displaying high or low levels of body weight loss [36]. AT *ALOX15* expression declines in the postpartum period, whereas *ALOX5* increases post-calving, and its transcription is enhanced in cows exhibiting higher levels of lipolysis [36]. Currently, the effect of ECS activation on bovine AT immune cells is unknown. In rodents, eCBs such as palmitoylethanolamide can polarize AT macrophages to the M2 anti-inflammatory phenotype when administered parenterally (SC, 30 mg/kg) for five weeks [100]. Considering the two-fold elevation observed in eCB levels during the postpartum period in cows with high weight loss [14], the altered gene expression of eCB oxidative enzymes between groups of cows exhibiting high and low levels of lipolysis [36], and the possible effect of eCBs on AT macrophage phenotype, the relationship between the ECS and AT inflammation should be further explored.

### The ECS regulates appetite and nutrient uptake

#### CB1 activation stimulates appetite

In mammals, CB1 increases food intake by activating the binding of orexigenic peptides and inhibiting the attachment of anorexigenic proteins to hypothalamic neurons [101]. Leptin, a key hormone in this metabolic equation, is released from AT after feeding and binds to the hypothalamus where it induces the release of anorexigenic peptides (extensively reviewed in [102]). Hypothalamic eCB levels are negatively controlled by leptin [103, 104], and disruption of leptin signaling leads to an increase in eCB expression in neural tissues. Hypothalamic levels of NAPE (an AEA precursor) increases after treatment with leptin in rats [104]. This pattern of NAPE expression identifies leptin as a potential suppressor of NAPE-PLD; this is reasonable considering it inhibits the mobilization of intracellular calcium necessary for the activation of NAPE-PLD [105]. In addition, eCB levels increase in the limbic forebrain of fasted rats and return to basal levels after feeding [106], further pointing toward leptin as a potential regulator of circulating eCB levels.

Peripheral CB1 activation may also exhibit an inhibitory role over leptin in neural tissue [107]. Studies by Tam et al. using a peripherally-restricted CB1 inverse agonist in a diet-induced obese mouse model reversed hyperleptinemia and leptin resistance [108, 109] and improved anorexic melanocortin signaling in the hypothalamic arcuate nucleus [110]. These findings highlight the capabilities of CB1 to reduce leptin sensitivity and satiation signaling pathways in the hypothalamus.

The role of the ECS on the hedonic response to eating has recently been described in detail [111]. Taste is of special interest as this sensation can be altered by endocrine and paracrine signaling [112]. In lean mice, the sweet taste is suppressed by leptin [113]; however, eCBs block this effect and enhance sweet taste sensation [114]. The capacity of eCBs to enhance sensitivity to sweet taste at physiological levels was described in humans, and, remarkably, taste response to sweet stimulation increased by more than 120% [115]. This was also observed in mice with a dose-dependent response [116]. In the same study, CB1 knockout or pharmacological inhibition obliterated the response to sweet tastes, suggesting that CB1 modulates the effects of this sensation. Furthermore, Rimonabant decreases food intake in mice, yet CB2-selective inhibitor SR144528 has no direct impact on appetite. Based on these findings, the involvement of CB2 on the modulation of appetite may not be as extensive as that of CB1 [117].

In dairy cows, reduced intake of feed promotes mobilization of body fat, which leads to increased hepatic deposition of TAG and synthesis of ketones. Although conjecture, by reducing ruminant AT lipolysis in a CB1-dependent manner, satiety signaling may be suppressed through the limitation of FA oxidation in the liver, subsequently increasing dry matter intake and improving periparturient metabolic health. For these reasons, the relationship between CB1 activation in AT and feed intake in dairy cows should be explored in the future.

#### NAPE-PLD, the intestinal barrier, and nutrient absorption

Recent studies determined that AT NAPE-PLD levels improve the gut epithelial barrier and microbial function, which in turn, enhanced AT energy storage function in a cyclical manner [118]. The intestinal epithelium regulates metabolic function through its role in the uptake of nutrients, secretion of hormones, and production of eCBs [119]. In monogastrics, short-term dietary FA contact in the stomach induces jejunal AEA mobilization and movement of FAs into the duodenum, which leads to enhanced synthesis of OEA [120]. In addition to the enrichment of eCB synthesis, activation of the gut ECS also improves adipogenesis in AT [121]. In monogastric species, the intestinal ECS reduces LPS mobilization, barrier disruption, gut inflammation, and dysbiosis of gut microbes [121].

In dairy cows, LPS released from the rumen epithelium is translocated across the intestinal barrier and into the bloodstream. Increases in plasma concentrations of endotoxin lead to profound metabolic changes and systemic inflammation [122]. In the same study, Ametaj et al. discovered that blood glucose and non-esterified FA levels correspond to circulating LPS [122], and such increases are accompanied by depressed dry matter intake in dairy cows [123]. Interestingly, local CB1 activation limits LPS absorption in the gut, which may improve appetite and limit inflammation in dairy cows.

## Conclusion

The past two decades of research have created a solid foundation in eCB biology with regards to the effects of ECS on the modulation of metabolic, behavioral, neurological, and immune functions in mammals. Targeting the systemic and adipose ECS shows promise to enhance periparturient period health through possibly promoting appetite, adipocyte proliferation, lipid accumulation, and suppressing lipolysis and AT inflammation. It is also important to determine the potential impact of ECS activity on fetal growth and neonatal health. Much work is still required to determine the biological significance of eCBs and ECS mechanisms of function in ruminant species but this research will help reducing periparturient disease incidence and enhace metabolic function in dairy cows.

## Data Availability

Not applicable.

## Abbreviations

2-AG: 2-Arachidonyglycerol
AA: Arachidonic acid
ABHD12: α/β Hydrolase 12
ABHD6: α/β Hydrolase 6
ACC: Acetyl-CoA carboxylase
AEA: *N*-Arachidonoylethanolamide
AKT: Protein kinase B
AMPK: 5′ Adenosine monophosphate-activated protein kinase
AT: Adipose tissue
CaMKKβ: Ca^2+^/calmodulin-dependent protein kinase kinase β
cAMP: Cyclic adenosine monophosphate
CB1: Cannabinoid receptor type 1
CB2: Cannabinoid receptor type 2
COX: Cyclooxygenases
CYP450: Cytochromes P450
DAG: Diacylglycerol
DAGL: Diacylglycerol lipase
eCBs: Endocannabinoids
ECS: Endocannabinoid system
ERK1/2: Extracellular signal-regulated kinase
FA: Fatty acid
FAAH: Fatty acid amide hydrolase
FAS: Fatty acid synthase
GLUT-4: Glucose transporter type 4
GPCR: G protein-coupled receptor
GPR55: G protein-coupled receptor 55
HSL: Hormone-sensitive lipase
LOX: Lipoxygenases
LPL: Lipoprotein lipase
MAG: Monoacylglycerol
MAGL: Monoacylglycerol lipase
MAGL: Monoacylglycerol lipase
MAPK: P42/44 mitogen-activated protein kinase
NAAA: *N-*Acylethanolamine-hydrolyzing acid amidase
NAPE: *N*-Arachidonylphosphatidylethanolamine
NAPE-PLD: *N*-Arachidonylphosphatidylethanolamine-specific phospholipase D
NO: Nitric oxide
OEA: Oleoylethanolamide
PEA: N-Palmitoylethanolamine
PGC-1α: Peroxisome proliferator-activated receptor gamma coactivator 1
PLC: Phospholipase C
PPAR: Peroxisome proliferator-activated receptor
PUFA: Polyunsaturated fatty acid
ROS: Reactive oxygen species
RXR: Retinoid X receptor
TAG: Triacylglycerol
TRPV1: Transient receptor potential cation channel subfamily V member 1
